# “Reply on: statistics on steroids—how recognizing competing risks gets us closer to the truth about COVID-19-associated VAP”

**DOI:** 10.1186/s13054-023-04351-7

**Published:** 2023-03-09

**Authors:** Fabiana Madotto, Amedeo Guzzardella, Vittorio Scaravilli, Giacomo Grasselli

**Affiliations:** 1 Dipartimento di Anestesia, Rianimazione ed Emergenza-Urgenza, Fondazione IRCCS Ca’ Granda Ospedale Maggiore Policlinico, Milan, Italy; 2 Department of Pathophysiology and Transplantation, University of Milan, Milan, Italy; 3 Department of Biomedical, Surgical and Dental Sciences, University of Milan, Milan, Italy

Dear Editor,

We read with interest Shorr and Zilberberg [1] about the association between ventilator-associated pneumonia (VAP) and corticosteroid treatment for COVID-19 respiratory failure. The authors wonder why three studies that analyzed this topic had conflicting conclusions. Indeed, we [2] and Lamouche-Wilquin et al. [3] documented an increased risk of VAP due to corticosteroid treatment, while no significant effect was detected by Saura et al. [4].

Shorr and Zilberberg hypothesize that the reason for differences lies in the analytical paradigms of the Saura et al. paper. In particular, Shorr and Zilberberg applaud the statistical approach (i.e., hazard ratio estimates) since capable of accounting for 1) the competing risks for VAP incidence (i.e., death) and 2) the non-proportionality of hazards over time (i.e., effect of corticosteroids treatment is time-dependent and it is allowed to vary as a function of time).

Regarding the first point, we would like to highlight that all the papers above accounted for competing risks but using different statistical approaches: Saura et al. performed a Cox regression with cause-specific hazard function, while we and Lamouche-Wilquin et al. used Fine-Gray sub-distribution hazard models. These approaches are equally valid and robust in competing-risk survival analysis. [5].

On the second point, Saura et al. assumed that the relationship between VAP incidence and corticosteroid treatment was not constant over time because the proportional hazard assumption for Cox regression was violated after a visual inspection of scaled Schoenfeld residuals. [6] Conversely, in our population, the same approach had not identified any violation of this assumption, and therefore, we decided to accept a constant hazards of VAP as a function of corticosteroid treatment. In Lamouche-Wilquin et al. paper, we did not find a description of accepting the proportional hazard assumption. Still, being this an a priori condition to carry out a Fine and Grey analysis, we assume it was tested and respected.

In summary, in all three studies, authors applied different—but equally effective and valid—statistical approaches considering the available data distribution, and all took into account the competing risk of death.

Therefore, we disagree with Shorr and Zilberberg that conflicting findings are due to different—and eventually inappropriate—statistical approaches. Instead, we believe that since statistical methods per se were adequate for all three studies, the different results are rooted in substantial clinical differences.

First, enrollment time frames were different among studies. We and Lamouche-Wilquin et al. included patients admitted to ICU from February to December 2020. In contrast, Saura et al. evaluated patients in ICU from March to May 2020, well before the publication of the RECOVERY trial’s results in June 2020 [7]. Consequently, the indications for corticosteroid treatment in the three study populations were dissimilar and not directly comparable.

Second, in our study, we included only patients treated with dexamethasone according to the RECOVERY trial and excluded those treated with delayed high-dosage corticosteroids (i.e., late unresolving ARDS [8, 9]). Similarly, Lamouche-Wilquin et al. included only patients treated with corticosteroids within 24 h after ICU admission, and most of their patients were treated according to the RECOVERY trial. Contrarily, according to the reported study limitations, Saura et al. included patients with heterogenous corticosteroid treatment. For example, the time between intubation and the start of the treatment was not explicated, which can explain why the Authors found a violation in the proportional hazard assumption. Moreover, use, type, and dosage widely varied among the study population. In other words, corticosteroid treatment for most of the patients in the Saura et al. report did not—and could not—comply with the available up-to-date evidence-based (i.e., the RECOVERY trial). In fact, just 25% of the patients included in the Saura et al. report were treated with dexamethasone. Notably, Saura and colleagues found a significant effect of dexamethasone on VAP incidence (*p* < 0.05) when they analyzed different corticosteroids separately.

In conclusion, we think that population selection affected these study findings more than the statistical approach used. Therefore, we are not confident as Shorr and Zilberberg in considering corticosteroids a small cost in terms of VAP. Instead,—as we previously concluded—clinicians should make every effort to implement protocols for the surveillance and prevention of infectious complications. Since the available evidence does not allow any firm deduction, further longitudinal studies could focus on the benefits and costs of DEXA connected to VAP incidence and survival.

## Data Availability

The datasets used and/or analyzed during the current study are available from the corresponding author on reasonable request.

## Abbreviations

ARDS: Acute respiratory distress syndrome
COVID-19: Coronavirus disease 2019
ICU: Intensive care unit
VAP: Ventilator-associated pneumonia
